# Alternative communication systems for people with severe motor disabilities: a survey

**DOI:** 10.1186/1475-925X-10-31

**Published:** 2011-04-20

**Authors:** Carlos G Pinheiro, Eduardo LM Naves, Pierre Pino, Etienne Losson, Adriano O Andrade, Guy Bourhis

**Affiliations:** 1 Laboratoire d'Automatique humaine et de Sciences Comportementales, Université de Metz, Bâtiment ISEA, 7 rue Marconi, 57070 METZ Technopôle, France; 2 School of Electrical and Computing Engineering, Federal University of Goiás, Av. Universitária, n. 1488 - Quadra 86 - Bloco A, 74605-010, Goiânia, GO, Brazil; 3 Faculty of Electrical Engineering, Biomedical Engineering Laboratory (BioLab), Federal University of Uberlandia, Campus Santa Monica, 38.408-100 Uberlandia, MG, Brazil

## Abstract

We have now sufficient evidence that using electrical biosignals in the field of Alternative and Augmented Communication is feasible. Additionally, they are particularly suitable in the case of people with severe motor impairment, e.g. people with high-level spinal cord injury or with locked-up syndrome. Developing solutions for them implies that we find ways to use sensors that fit the user's needs and limitations, which in turn impacts the specifications of the system translating the user's intentions into commands. After devising solutions for a given user or profile, the system should be evaluated with an appropriate method, allowing a comparison with other solutions. This paper submits a review of the way three bioelectrical signals - electromyographic, electrooculographic and electroencephalographic - have been utilised in alternative communication with patients suffering severe motor restrictions. It also offers a comparative study of the various methods applied to measure the performance of AAC systems.

## Introduction

Much research work has been devoted in the past twenty years to developing assistive technology (AT) devices aiming at offering to people suffering a motor disability of various origins (e.g. locked-in-syndrome, amyotrophic lateral sclerosis, quadriplegia, muscular dystrophy, cerebral palsy, etc.) associated to disorders of verbal communication, the possibility of communicating with the persons in their entourage and having some control on their environment. These AT devices are operated by human-machine interface sensors receiving information provided by the person with disabilities to pilot a graphical user interface [1].

When working in the area of augmentative and alternative communication (AAC), one of the recurring problems is selecting the sensor that will be best suited to the user's motor capacities, whatever the type of AT devices (communication aid, assistance when using the computer, etc.) used. As a consequence, one of the first tasks to be done is identifying the proper sensor from among the set of devices available on the market or developed in research labs.

One of the major difficulties encountered in the quest for a well-adapted AT devices is that the selection process is strongly influenced by the user's specific needs, which in turn has an impact on the type of sensor to be used. Thus, this process cannot be carried out without taking full account of the human-machine system to which it is going to be applied. It is therefore necessary to study the performances of the user-sensor-system trio.

The purpose of this paper is to report about our study regarding the several technologies employed in the restricted area of alternative communication systems based on bioelectricity. The first part covers the main types of bioelectrical signals used as control sources in modern AAC systems, notably the electromyogram (EMG), the electrooculogram (EOG) and the electroencephalogram (EEG). The second part offers a review of the various methods described in the literature to measure the performances of the communication aid devices.

## Switch based control and Proportional Biosignals sensors

A human-machine interface sensor can be defined as a device meant to capture and transmit the action that the user intends to perform. Due to the wide range of ways for the sensors used as communication aids to capture and transmit information, it appears necessary to sort them according to the operational mode. Two function types can then be identified: the switch-based control (SBC) sensor type and the proportional sensor type (PRO). This classification is no obstacle to the possibility of combining these functions and thus raises the number of possibilities of interaction when using the communication aid. For instance, sensors designed to act as a mouse replacement call on these two function types with the click button (SBC) and the movement controlling a cursor (PRO) on the screen. A SBC type sensor just transmits binary signals to the AT device, whatever technology has been applied for its conception. Consequently, this device is the least efficient when used for interaction with a communication aid. Such systems work only as a tool to scan various possibilities and make a selection, the major difficulty being then to decide about the scanning delay [1]. The PRO-type sensor has the clear advantage of representing several binary commands using only one proportional signal in the same way one integer number is represented by one or more bits, thus saving the user time and effort during the AT device operation. However, these sensors - that are generally used to control a cursor on the computer screen - suffer a drawback related to their mode of operation: the person with disability is supposed to have sufficient dexterity to control them, which is not always the case.

To take advantage of an electrical biosignal in AAC tasks, the user may express his intention in three different ways: eye movements (EOG), muscle (EMG) and cerebral activity (EEG). Even though these sensors may also be used to provide a progressive signal as in [2], they usually deliver a binary one, which demands lesser control over the body functions by the user.

One example of using the biosignal with either PRO or SBC type sensor is the interpretation of the EMG signal. The envelope amplitude may be translated to: (i) a binary signal, when compared against a threshold (muscle contracted or not) or (ii) a continuous signal scaled between 0 and a maximum value. It must be observed that the use of the progressive signal in this specific case is highly dependent of the final application, which may be more or less susceptive to envelope amplitude variations. Another case is using the EOG signal to deploy a PRO-sensor, as each degree of eye movement represents changes from 14 to 16 μV in the recorded signal.

Other strategies generate discrete signals employing binary information obtained from a biosignal. Barreto et al. [3] controlled a cursor that was allowed three different levels of speed, which increased over time as long as the muscle remained contracted.

The biosignal sensor is generally more complex than those using mechanical principles, requiring elaborated circuitry for data acquisition and signal processing algorithms. In spite of the technical feasibility of exploring electrical biosignals, the application of those kinds of sensors for alternative communication is indicated mainly in cases of severe motor disabilities. In situations where the user demonstrates the ability to move some part of the body, other approaches will probably be more suitable than using electrical biosignals: (i) head movements tracked by a camera is an alternative to control a cursor using EMG from facial muscles; (ii) sensor using mechanical principles, such as pressure membrane based devices activated with the tongue [4] or the side of the head are easier to operate and less prone to errors than a BCI system.

## Electromyography

### EMG signal

When the brain commands a muscle to contract, signals are sent to motor neurons that in turn, control several fibres. As the membrane fibre is depolarized, an electrical potential is generated in the vicinity of the muscles fibres with duration of approximately 8 ms. The summation of the action potentials propagating trough the fibres yields the motor unit action potential (MUAP) of a motor unit (MU). To maintain the force exerted by the muscle, the MUAPs are fired repeatedly, with frequency from 7 to 20 Hz [5], forming the sequence known as MUAPT (MUAP train). The electrode used to record muscle activity will register the electrical fields generated by all the motor units in the range. This ensemble of MUAPTs is the EMG signal. Recorded at the skin surface, the EMG signal may present 20-2000 μV peak-to-peak amplitude values [6].

There are two kinds of electrodes for EMG signal acquisition: intramuscular and surface electrodes. The former type is preferred for clinical applications, as in the diagnosis and evaluation of motor diseases [7]. Information from specific motor units or even fibres can be acquired with confidence; however it may produce infections and the mechanical action into the muscle may cause lesions. Consequently, the choice on using surface electrodes is appropriate for applications such as communication aid devices, as they will probably be used for several hours a day.

The most common electrode type is the Ag/AgCl, usually 1-3 cm in diameter. Before the application of the electrode, the skin is cleansed with alcohol-wet swabs and a conductive gel is used to increase conductivity. One of the concerns is that conductivity decreases and thermal noise increases as the gel dries off.

While the majority of studies referenced in this article reported the use of Ag/AgCl electrodes, some omitted the information and only one [8] reported the utilization of a noncontact electrode that could be used over clothes, however no technical details are provided.

Electromyography is the process of recording and analyzing the EMG signal. One of its main applications is making a diagnosis and assessing the severity of the disability in case of neuromuscular disorders. Another application is the possibility of identifying the strategy used to control skeletal muscles during a movement, so it is later employed to operate prosthesis. Yet another approach consists in using the EMG signal as a source of information to control devices such as electrical wheelchairs [9]. The number of studies exploring the EMG signal potential for AAC is considerably lower when compared to the attention devoted to other possibilities such as EEG signals.

The EMG signal, used as a channel for AAC, is usually acquired with sampling rates in the order of 1 kHz. This aspect is particularly important when spectral tools are employed, considering the Nyquist theorem and that most part of the signal energy is presented up to 500 Hz. Even though, in some studies, the sampling rate may be lower. The reason is not explicit, but could be connected to the necessity of the system to operate in real-time, which could be complicated by a large amount of data generated by higher sampling rates.

The algorithms used to process the EMG are usually simple, especially when operating as a SBC sensor. The translation into a binary variable calls for simple strategies - related to signal amplitude - such as the variance of the root mean square (RMS) value of the signal [10]. The spectral domain analysis is also analysed. Signal features such as mean and median frequency (MNF and MDF) can be used to define when a muscle is active as their value shifts during contraction. The spectrum can be divided into sub bands before extracting the features [3,11-13]. In [13,14], as various muscles typically supply different MNFs, muscle activity can be correctly detected, in spite of interference due to the activity of others muscles.

### EMG applications

Communication aid devices using EMG signals can be subdivided into three major groups: (i) emulate mouse, (ii) speech recognition and (iii) act as a switch-based control device. Most of those applications use the SBC sensor approach with more complex sensors deployed by using more than one muscle at a time.

#### Switch-based control device

In situations like the locked-in syndrome, the patient will present control of eyes and cognitive tasks. Therefore, an easy way to communicate is by answering to "yes/no" questions. This approach provides an output channel of communication with low transfer information rate, low interactivity and the care provider must have the necessary skills to formulate the right questions.

Patients that are still able to carry out residual movements with one of the limbs, or to move their head, may use a pressure device. The same principle can be applied to EMG signals where the pressure action can be replaced by muscle contraction. Electromyography has already been considered as a way of assessment for patients with disorders of consciousness, indicating its use as a channel of communication [15]. Using computational solutions, one can generate binary signals, with '1' being associated to muscle activity and '0' otherwise.

One approach consists in composing messages with some kind of code - e.g. the Morse code. Even if it is an unnatural mode of communication, the procedure might turn out to be extremely valuable for persons with severe motor impairments. Any biosignal which can be interpreted as a two-state information source is a potential candidate to use such code. From the Morse-based code the user can control devices and communicate in several ways, depending on the strategy adopted. Studies using other sources than the EMG signal have shown that applying the Morse code [16,17] can be a good option for AAC. As using the Morse code is not natural task in terms of language and that keeping a typing rate is difficulty for the persons with disabilities, modified forms of the code may be adopted [16].

Park et al [18] developed a system where the user moves his chin so the signal acquired from the *Massetter *muscle is transformed into "dot" or "dashes" symbols depending on the duration of the contraction. The sequence is decoded into characters that in turn feed a voice synthesizer. This study includes a method for fatigue adaptation, an important concern when using EMG. The major caveat was that people are not capable of chew-and-pause fast, so the transfer information rate was low, although not reported in numbers.

The binary signal can also be used to operate a scanning device, e.g. a virtual keyboard and some more complex interfaces. The Impulse™ [19] system - one of the few AAC commercial solutions based on the EMG signal - uses this approach in a wireless solution to offer computer access with a specific scanning interface.

When comparing the scanning and the code-based approaches, we can observe that both are based in simple signal processing techniques. The difference lies in the cognitive effort required from the user: the scanning approach transfers the complexity of the message generation process to the system interface whereas, with code-based devices, the user has to learn the sequence of symbols necessary to compose each character or command.

#### Mouse emulation

Since the 1970s the mouse is, along with the keyboard, the standard input device for computers operation. Then, it is understandable the number of studies to develop devices that provide mainly point-and-click functions as a physical keyboard can be replaced by a virtual one. For some studies, even though the goal is to provide a hands-free alternative to healthy people, the solution could be adapted for the person with disability.

Using muscles in the pointing task can be described as a three-step process: (i) identifying the suitable muscles to be explored and based on this, (ii) define what kind of control can be obtained and (iii) last, choose and process the EMG signal feature to generate the command.

In the case of people with severe motor disabilities, facial muscles are a common option as they can be activated, even in the case of people who suffered a severe spinal cord injury.

Once the number of muscles available has been established, research can start to set up the strategy to achieve proper cursor control. One simple strategy is to use each muscle to define the cursor displacement in one direction. There are at least two aspects that will define the system final capacity to control a cursor: the sensor type and the user ability to control the muscles. If a PRO sensor is used and the user has the dexterity necessary to control all the four muscles at once, the cursor control will be omnidirectional. If an SBC sensor is employed with the same user, than the cursor can moves towards eight different directions. Finally, if the SBC sensor is operated by someone with poor muscle control, it is likely that the cursor will move to only four different directions. The most commonly used facial muscles in the pointing task are the *Corrugator*, the left and right *Frontalis*, the left and right *Temporalis *and the left and right *Zygomaticus major*. Traditional approaches use pairs of muscles to control displacement over the XY axis. Others strategies for exploring the EMG signal are possible, such as the 2D control from only one [20] muscle.

Considering all the actions a mouse can perform there are also right and left-clicks. In [3] the left-click action and cursor movement (2D) were controlled by the EMG signals from the *Temporalis *and *Frontalis *muscles. Additionally, the system is provided with an ON/OFF switch, controlled by the EEG signal. The mouse functions were identified applying a threshold to the amplitude signal, and later, performing spectral analysis over several frequency bands. The system was implemented over a DSP board, which was identified by the host as an ordinary mouse. The final command information generated from the EMG signals was not actually continuous as the application may suggest. Actually, the system used a three-level adjustable speed schema, with increasing values if the system identified five consecutive commands indicating the same direction. At first, only three channels for EMG signals were necessary, but in [12,21] a fourth electrode was used, improving the average of correct classification of muscle movements from 78.43% to 98.42%. Despite the good results for classification muscle movements, the system took 16.3s in average to move a cursor from the middle to the corner of the screen, compared to 1s-2s with a standard mouse. So, in [13,22,23] the same approach was combined with a gaze-based system into a hybrid system. While the gaze offered the absolute position of the cursor, the EMG signal provided incremental displacement. As consequence, the time to move a cursor from the middle to the corner of the screen dropped from 16.3s to 6.8s.

Also considering multimodal approaches, a detailed study [24] compared a standard mouse and a hybrid device. The cursor position was controlled by gaze and the object selection (left-click) was activated by frowning. The two solutions were compared using Fitts' law: for small distances a standard mouse showed superior performance, but there was no statistical difference among devices with large distances.

In [25] the goal was also to provide a pointing device controlled by facial muscles. A continuous Wavelet transform measured the level of activation of each muscle, providing four direction displacements, associated with both sides of *Orbicular, Massetter *and *Mentalis *muscles. Left and right click operations were associated to opposite directions executed at the same time (Up+Down = right click and Left+Right = left click). The use of the Wavelet transform was justified by the shape similarity between the wavelet mother and the MUAPs. However, this strategy can be questioned, as the system performance was not compared with traditional signal features (e.g. the RMS value). Even the similarity between the Wavelet mother and the MUAPs can't be assured, as no other arbitrary wavelet mother was used and the electrodes dimensions were not reported, being impossible to estimate the electrodes selectivity regarding muscle units.

Using four muscles to control horizontal and vertical displacement seems like a very straightforward idea. Nevertheless, in a novel approach [20] the authors employed only one muscle to control the cursor position in the X and Y axis. The power levels of two different frequency bands extracted from the EMG signal recorded from the *Auricularis Superior *muscle were employed. The strategy adopted is quite different from the others described earlier, as the absence of muscle activity sets the cursor the position to coordinates (0,0) while the contraction moves the cursor. The user training is mandatory, as not only the user should learn how the contractions affected the cursor position, but also because of the different bands of interest presented by different users.

For patients unable of controlling upper limbs, using muscles located in the head may be the only option. However, people with conditions such as tetraplegia may manifest residual control of neck, shoulders and even arms.

Additionally, using facial muscles seems as an unnatural way of controlling a cursor, when comparing to a standard mouse. As example, diagonal movements tend to be accomplished through horizontal and vertical movements [26] when using EMG signals as source of control. Head motion [27], on the other hand, could be compared to a joystick operation.

In [28,29], five different motions of neck and shoulder could be recognized with 95% mean recognition rate and response time about 0.17s. Two pairs of electrodes were placed over the *Sternocleidomastoid *and the *Trapezius *muscles, in each side of the body.

In [26] three methods offering pointing device control were compared: a standard mouse, head-orientation using an accelerometer and the EMG based approach. The *Platysma*, left *Trapezius *and the *Frontalis *muscles were utilized. Cursor speed was a continuous variable, with a maximum value attributed to 70% of maximum voluntary contraction (MVC). As expected, the mouse was superior and in general the EMG approach was inferior to the head-orientation method, especially due the difficulty to perform diagonal movements.

In [30] the angle of head was estimated through linear interpolation of the EMG signal extracted from the *Sternocleidomastoid *muscle. For small angle rotations the EMG signal is too small to offer any useful information and in its place a camera was used and the angle was estimated by the relative position of the pupils. In fact, if the user presents good head and neck control, the camera based solution seems to be more appropriated, with software already available for download, demanding only an ordinary webcam.

Finally, there is the possibility to use movements of arms to operate virtual keyboards and mouse. In [31] an omnidirectional pointing device is controlled by the EMG signals recorded from the forearm. An Artificial Neural Network (ANN) was used to find the direction, while the muscular contraction level controlled the cursor velocity. A recent research involving the Microsoft Corporation presents a similar approach using the EMG signal in games [32] interfaces but also in hands-busy situations, that could also be deployed for people presenting some level of disability. The implication of a company highly bounded with the computing area indicates the potential of using the EMG signal for computing interface. However, solutions for user presenting good arm movement control are outside the scope of this article and even in the case of adopting an assistive device, adapted mouse or joysticks would be more appropriated for this user profile.

It was noticed that some studies lack a method to measure performance, impeding therefore the comparison of different approaches. Fitts' Law was already used to compare different pointing devices [33] and has been used in several studies; precision on drawing over templates are also suggested [31]. The methods used for performance measure are presented in details in section 6. Other problem with pointing devices studies is that details such as screen resolution and specifications of the standard mouse used are not revealed.

#### Automatic speech recognition (ASR)

Since the late 1960s, efforts have been made to achieve a system for speech recognition [34]. Several pieces of software are available in the market and modern operating systems for personal computers already offer built-in speech recognition. But there are a few drawbacks that offer some resistance for using usual ASR systems and intensify the research on silent speech interfaces: (i) the audible speech prohibits confidential conversation; (ii) it is not advised to use such systems during meetings or inside a library; (iii) the performance decays severely in adverse environments such as crowded places; and finally (iv) some clinical conditions hinder voice communication.

There is a relation among the words pronounced and movements of articulatory facial muscles. Then, a feasible approach is to use the activation of those muscles to identify phonemes and therefore, words. This is not an easy task, as the act of speech employs several facial muscles, such as: *Mentalis, Depressor anguli oris, Massetter, Digastric, Zygomaticus major, Levator anguli oris, Platysma, and Orbicularis oris*.

Studies usually regard people with voice impairment, such as conditions after a total laryngectomy [35] or to situations where the ambient noise impedes communication (e.g. by fire fighters and pilots). Therefore it must be carefully analysed if the level of disability may compromise the control over the muscles involved in the speech process. Nevertheless, people with severe motor impairment could use ASR by muscle activity to achieve a channel of communication. One example is people with tetraplegia using ventilator systems that are adjusted to accommodate cardiopulmonary requirements, but that are not optimal for speech. Speech produced with typical ventilator adjustments is often characterized by short phrases, long pauses between phrases, abnormal loudness, and poor voice quality [36]. In a study conducted by Denby et al. [37] over silent speech interfaces - systems enabling speech communication when an audible acoustic signal is unavailable - several solutions are compared regarding if the systems: (i) are invasive, (ii) work in noisy environments, (iii) require glottal activity, (iv) are ready for market, (v) work for laryngectomy and (vi) have low cost. Among the seven systems analyzed, the EMG based had the highest overall evaluation.

In [38], Hidden Markov Models were used to map muscle activation into phonemes. The features extracted from the EMG signal were mel-frequency cepstral coefficients (MFCC), as previous studies showed that discrete wavelet transform (DWT) coefficients were superior but slightly different. Only three channels were used with respect to the muscles *levator anguli oris*, the *zygomaticus major*, and the *depressor anguli oris*. The muscles used and the electrodes were defined heuristically. To evaluate the system a limited vocabulary of 60 words was used and accuracy of up to 85% was achieved.

In [14] a multimodal ASR with the acoustic information and the EMG signal allowed a Coupled Hidden Markov Model (CHMM) to recognize speech. This solution is compared with two others: audio only and EMG only. Adding different levels to the signal, it showed that the audio-only approach is highly dependent on the SNR, while the EMG-only proposal was not affected. Five muscle channels were used: the *Levator anguli oris*, the *Zygomaticus major*, the *Platysma*, the *Depressor anguli oris*, and the anterior belly of the *Digastric*. No criteria were indicated for choosing neither muscles nor the electrodes position. The vocabulary used was extremely restricted, with only 10 words.

In [39] only vowels were used as shape of lips and mouth cavity were stationary. Three channels were used, with information recorded from the *Mentalis, Depressor anguli oris *and *Massetter *muscles, since those are the most active muscles during vowels pronunciation. An artificial neural network (ANN) using the back-propagation algorithm was used to associate the RMS of the EMG signal with the vowels. Other studies provide the recognition of isolated words and small vocabulary [40-42]. In [42] as the aim was to recognize speech of pilots that could be interpreted as commands, electrodes were embedded in a pilot oxygen mask. The error rate was very low, ranging from 0% to 10.4% during the task of recognizing the speech of the numbers 'zero' to 'nine'.

The probability that electrodes are repositioned in the same place as the previous session is very low. In [40] a normalization method found that among sessions, the accuracy to drop about 10%, whereas without the method the accuracy dropped more than 21%. Eight channels of information were extracted from the following muscles *Levator angulis oris*, the *Zygomaticus major*, the *Platysma*, the *Depressor anguli oris*, the anterior belly of the *Digastric *and the tongue. Only the numbers 'zero' to 'nine' composed the vocabulary.

Wand et al. [11] and Jou et al. [43], implemented continuous speech recognition using an HMM algorithm. The vocabulary was phonetically balanced and formed by 108 words. A total of six channels were used. When compared to features from frequency and time-frequency domains, the Wavelet transform showed a slightly advantage.

Studies show good results as almost 90% of accuracy is obtained. On the other hand, the vocabulary used is usually extremely restricted. But as it happens with other categories of assistive technology devices, people with severe motor impairments may find even a limited control extremely useful. If 60 words could be associated to different actions, common sense dictates that even this would be extremely helpful for daily activities.

Other issue is that the data used to test each system were obtained under highly controlled situations, with the subjects being under supervision. During normal operation, the user will probably be less concentrated, the pronunciation sometimes will be less clear and the system may not respond very well.

As the goal is to associate phonemes with activation of related muscles, it is interesting to define some criteria to decide which muscles should be used, as well the electrode positioning minimizing crosstalk. Although not applied to ASR, in [44] the assessment was conducted for better positioning of electrodes in the forehead, so both electrodes could gather information from different muscles with minimum interference.

## Electrooculography

### EOG signal

The EOG signal is the electrical signal generated by the difference of potential between the cornea and the retina, from 2 to 20 mV but with recorded signals ranging from 15-200 μV [45]. This potential is due to the large presence of active nerves in the retina compared to the front of eye [46]. Several experiments show that the corneal part is a negative pole while the retina is a negative one in the eye. Then, analyzing the eyeball as a dipole eye movements can be registered through the EOG, with each degree representing 14 to 16 μV in horizontal and also vertical way. Sampling rate for electrooculogram acquisition should reproduce components up to 15 Hz [45]. Traditionally, for EOG recording are placed five Ag/AgCl self-adhesive electrodes: (i) one pair above the eyebrow and below the eye to record vertical movements; (ii) one pair next to the lateral canthus, to record horizontal movements and (iii) one over a neutral site, acting as reference.

### EOG applications

Compared with the EEG, EOG signals have the characteristics as follows: the amplitude is relatively high, the relationship between EOG and eye movements is linear, and the waveform is easy to detect [46]. Considering the simplicity of EOG is also easier to classify it when compared to EMG. For these reasons, EOG-based HCI systems have become a very interesting field of research in recent years. In addition, the majority of the patients with severe motor disabilities remain able to control their eye movements. In this sense, recent studies have shown the viability of the EOG application in assistive communication systems.

Borghetti et al [47] developed a system for writing in an alphanumeric matrix based on two EOG channels (vertical and horizontal). The cursor movement in the orthogonal directions was carried out by EOG classification based on elementary parameters like polarity, amplitude and slope, and the letter selection was made by double blinking detection from EOG. The study appealed to the low cost, around € 100. As a preliminary research, there were no bindings with voice synthesizer or other software of any kind. The interface was very simple as even the backspacing function was missing.

Usakli et al [48] proposed a similar system where both cursor movement and letter selection were supported by an EOG classification algorithm based on the nearest neighbourhood (NN) relation. The performance of the designed system was compared with that of a P300-based BCI speller. Results showed the EOG system more efficient than P300-based BCI system in terms of accuracy, speed, applicability, and cost efficiency.

Dhillon et al [49] proposed a virtual keyboard writer system based on two EOG channels and one EMG channel. The cursor movement was associated to the gaze angular displacement in the vertical and horizontal directions, and the letter selection was carried out by an "EMG click" obtained from eyebrow. The authors reported as advantages the lower cost and complexity of their system compared to more sophisticated methods to detect eye movements like videooculography (VOG) and infraredoculography (IROG).

Other studies prove the possibility of using eye movements as a control source, such as in [46] to control a mini-car. The signal was transformed into trains of rectangular pulses and moving the eyes twice in the same direction indicated the command the mini-car should execute. A feedback was offered to the user, allowing the command confirmation, also through the eyes. Other applications demonstrating the potential of the EOG signal for control are to handle wheelchairs through eyes movements [50-52] or to control a robot [53]. Cursor control was showed in [51] where a simulator on the screen was used to train the patient to control a powered wheelchair and in [53] to control a robot. Being a manifestation of eye movements, EOG signals are processed to identify gaze [50,51,54,55], usually for cursor control on the screen. However, the most popular method for gaze estimation is using infrared cameras [55], through the reflection in the eye structures and their geometric relations. In fact, gaze based devices may be best suitable for users with severe motor impairment that cannot move the head, as one of the technical problems presented by gaze based devices that is the lost of reference once the user moves it. Cursor control may be offered with absolute coordinates (gaze) [54,55] or through direction of movement [56,57].

With a simpler application, EOG signal may be used to encode Morse code messages [58] with looking left and right as 'dash' and 'dot', respectively. Another way of communication is to associate eyes movements sequences with symbols [59] that in turn, could be characters or even high level commands.

## Electroencephalography

For people with severe motor disability such as locked-in syndrome, it often becomes impossible to communicate or control a muscular activity. However, these people generally keep cerebral and sensory functions intact. A solution planned to overcome this handicap is to use electroencephalography associated with the cerebral activity to control an interface. This type of interface using the cerebral waves is usually called brain computer interface (BCI). The approach used to build a BCI consists in measuring the cerebral activity through the EEG signal in order to determine the wishes of each subject. BCI applications can also be built with invasive technologies, e.g., electrocorticogram (ECoG), which involve implantation of electrodes in the cortex and provides better signal to noise-ratio [60]. Although invasive technologies are suppose to deliver higher-dimensional control, studies conducted by Wolpaw and McFarland [61] have shown that non-invasive EEEG-based BCI can give multidimensional movement control comparable to the control achieved by invasive BCIs. As invasive methods face technical difficulties and involve clinical risks, they should only be used in rare circumstances, when they are necessary to avoid artefacts (e.g. uncontrollable head control) or in cases where the invasive solution shows clearly superior performance than non-invasive methods. For those reasons, we focus on non-invasive BCI applications that use EEG.

Of course, it is not possible to analyze complex thoughts but to detect for example variations of rhythms associated with sensorimotor activities. Thanks to the analysis of this type of EEG signals associated with an imagined motor activity (sensorimotor rhythms), it is possible to build interfaces in which the displacement of a cursor present at the screen (according to one or 2 dimensions) is controlled in a continuous way by cerebral waves [62].

Other EEG signals associated with visual or auditory stimulations allow the construction of brain computer interfaces; these signals are called the evoked potentials. The P300 waves are cognitive evoked potentials often used for BCI. For interfaces based on the treatment of P300, it is not allowed to provide a continuous control but to choose one item among several [63]. Contrary to the first case where the subject has to modulate EEG rhythms in a spontaneous way, in this last type of interface the evoked potentials are created by stimulations of the interface on the user, this strong dependence between stimulations and interface explains the term synchronised interfaces used.

It is thus possible starting from the two examples of EEG signals presented to distinguish two types of BCI:

• synchronous interfaces based on evoked potentials;

• asynchronous interfaces based on EEG signals obtained in a spontaneous way by the subject, the sensorimotor rhythms being the most usually kind of EEG signals used in this type of interface.

Each one of these two approaches present performances, advantages and disadvantages which will be presented in the following paragraphs.

### Asynchronous BCI

Some asynchronous BCI use slow cortical potentials (SCP) corresponding to shifts of the mean potential measured on the cortex. Relatively long recording times (several seconds) are required before being able to discriminate between a positive and a negative shift of these potentials. A negative shift of the cortical potentials is associated to a significant cortical activity (imagination of movements or mental tasks) while a positive shift corresponds to a reduced activity. The main advantage of the use of SCP is the relative simplicity of the processing of these EEG signals, requiring only filtering and artefacts corrections. The major disadvantage is related to the need for more or less long training before the user is able to control the interface with an acceptable accuracy. The most well known application using SCP is the TTD developed at the University of Tübingen in Germany [64]. From this TTD, an internet navigator called NESSI was developed [65].

Another kind of asynchronous interface can be obtained from the processing of sensorimotor rhythms Mu and Beta associated to the imagination of the movements of the right and left arms or hand for example. The interfaces built from sensorimotor rhythms use mainly cursor displacement on the screen controlled by the magnitude of Mu and Beta waves associated to the cerebral activity in the sensorimotor cortex. The Mu waves frequency range is between 8 and 12 Hz and the Beta waves frequency range is between 18 to 26 Hz. These waves present interesting properties for BCI because they are associated to region of the cortex directly connected to the control of motor activity. Any preparation of a movement accomplished or imagined by the right arm or hand, respectively left, result in a decrease of the amplitude of these Mu and Beta waves detected on the left hemisphere, respectively right, of the sensorimotor cortex. This decrease of amplitude of the Mu and Beta rhythms is due to the desynchronisation of neuronal activity in the sensorimotor cortex of the hemisphere opposed to the requested arm or hand, this phenomenon is called ERD for Event Related Desynchronisation [66]. The change in amplitude or energy contained in the Mu and Beta bands is the feature that must be extracted from EEG signals. This one can thus be quite simply obtained by estimating the energy of the signals obtained by two band pass filters, one covering the Mu band and the other the Beta band [66].

Another procedure consists in making an autoregressive frequential analysis in an adaptive way in order to extract relevant parameters continuously with a greater speed [67]. After this step of feature extraction, classification is generally necessary to discriminate between the two classes (right-hand side or left) starting from the parameters which it was possible to extract from the signals. Studies showed the good robustness of the linear discriminating analysis in this type of study [68].

A continuous value is obtained from classification, and from this value, it is possible to control the interface in real time. The interface is thus controlled in a continuous way, allowing applications like the displacement of a cursor or an object on a screen. Several alternative communication interfaces use this approach to carry out the selection of item by controlling the displacement of a cursor towards the target corresponding to the choice of the subject [69,70]. A continuous control is even much more important in applications where the subject must control prosthesis or the displacement of his wheelchair [71,72]. These applications require, in addition to continuous control, a good accuracy which is generally the case for BCI based on sensorimotor activity.

Another advantage of the use of the Mu and Beta rhythms comes from the relatively good localization of them on the cortex, which makes it possible to consider applications based on only 2 EEG sensors [73].

However the major disadvantage of asynchronous BCI like those based on the sensorimotor activity is the need for relatively long training sessions before being able to control the interface accurately.

Insofar as these brain computer interfaces short-circuit the defective transmission of information between the brain and the muscles, it is possible to imagine building interfaces ensuring a faster transmission and thus acceleration in the execution of orders of the brain. However, in practice, it is for the moment difficult to obtain higher performances in term of speed and reliability of transmission starting from brain computer interfaces. Experiments comparing times of positioning one cursor on the screen of a computer starting from a joystick and starting from the EEG signals coming from the sensorimotor cortex show the advantage in term of speed and accuracy of the use of the joystick [74]. Results of works on brain computer interfaces based on sensorimotor EEG signals show the very strong variability of the performances as one of the main difficulty to overcome [75]. Indeed whatever the measures (EEG or ECoG), the methods of analysis of the signals, the studied subjects, it always remains a significant problem of performances reproducibility. The origin of this problem is still difficult to define, for some researchers it is not due to the methods of measurements and analysis, in spite of the progress obtained following many works during the 20 last years, but more surely due to the higher difficulty for someone to control his EEG signals coming from the sensorimotor cortex than to control his muscles [75]. Relatively long training sessions are necessary to control EEG signals, this training being done by a visual feedback of the tasks carried out on the screen of the computer. Nevertheless it remains difficult, including for the most gifted subjects, to reproduce with the same reliability and the same speed of the actions carried out by our muscles starting from the control of our cerebral activity in the sensorimotor cortex.

### Synchronous BCI

In this type of interface, EEG signals used are not created in a spontaneous way by the user but are synchronised on stimulations sent by the interface. These stimulations are generally visual and more rarely auditory, and EEG signals are generally called evoked potential. Among the evoked potentials, those which are generally used to build a BCI are the SSVEP and P300.

The wave P300 corresponds to a short positive deflection of the EEG signal which appears 300 ms after a rare and awaited stimulation within a great number of stimulations [76]. A well known experimental design used to obtain this P300, is referred to as the oddball paradigm [77]. A communication interface based on this paradigm, which makes it possible to select letters of the alphabet to spell words, is called P300 speller [78]. This P300 speller is generally formed by a 6 × 6 matrix containing 36 items (letters, numbers or characters) which it is possible to select. The experimental procedure consists in flashing successively each line and each column in a random way. The selection of one item is carried out by the detection of the P300 which will appear in response to an awaited stimulation which is the flashing of the line or the column containing the desired choice. These awaited stimulations are rare (2) since they appear in a random way among the succession of the flashing of each line and each column of the matrix (12: 6 lines and 6 columns). The detection of the P300 is generally made by carrying out averages on a great number of stimulations, because these waves have very low amplitudes if one compares them with sensorimotor rhythms for example. Another difficulty to detect these P300 quickly lies in the fact that they are not as well localized as sensorimotor rhythms on the cortex, it exists indeed several cortical centres of the wave P300 [76]. The minimum number of electrodes to be used in the case of an interface containing P300 will be thus a priori higher than that necessary for a BCI based on sensorimotor rhythms.

In spite of the difficulty of measurement and analysis of the P300, many BCI based on this type of wave were made, P300 speller being most known [79,80]. The major advantage of this type of interface lies in the absence of training necessary to the subject to control EEG signals since those are stimulated by the interface. Nevertheless, offline analysis of training session is generally needed in order to optimize the algorithms used to process the P300 which enormously vary from one person to another but also on the same subject according to its state of tiredness. The adaptation effort is thus deferred subject towards the software.

Performances records in term of speed thus could be carried out on P300 speller [81]. In best case, the time needed to select a choice is of the same order of magnitude as that necessary to a subject having to move a cursor on a precise place of the screen with a mouse. These results let consider the possibility of fast information transmission through BCI systems. Nevertheless studies on the reproducibility and the reliability of these types of communication systems must be done, because the significant effort of concentration that is requested from the user involves rather quickly a tiredness of this one. Recently a commercial BCI became available: the Intendix^® ^[82] is a typing device that uses visual evoked potentials running over a non-dedicated personal computer.

The other evoked potential often used for BCI is the visual evoked potential and in particular the SSVEP. This evoked potential is recorded on the visual cortex in the occipital lobe. Contrary to P300, this evoked potential being better localised on the cortex, only 2 or 3 electrodes are necessary. The stimulation which allows the appearance of a SSVEP is induced by a flickering at a frequency higher than 6 Hz present on the target which the subject must gaze [83]. It is thus possible to build an interface containing different items which one associates different frequencies of flickering; the detection of item is carried out by a spectral analysis of the EEG signals which must reveal the frequency of the target that the subject gazed. The number of target may be from 4 to 48, the range of the frequencies usable from 6 to 24 Hz and the resolution given for these SSVEP is of 0.2 Hz [83]. The use of SSVEP makes it possible to obtain information transfer rate of 46bits/min with an accuracy of 95% [84]. Just like P300 speller, this type of interface requires an increased concentration of the subject, which lets predict a significant tiredness for the user.

### Considerations about synchronous and asynchronous BCIs

The use of cognitive evoked potentials appears to be very interesting to make communication interfaces for people with severe disability; the principal reason is the absence of training requested from the subject. However, a lot of concentration for the subject and relatively powerful measure and processing systems are necessary; this can explain why no low cost synchronised BCI has been developed. On the other hand, there are systems at more accessible prices which use and treat sensorimotor EEG signals for bio-feedback or video games. Another interest of the use of the sensorimotor rhythms lies in the possibility of continuously control; moreover the systems having an asynchronous control are used in a more natural way by the subjects.

The debate on the choice of the type of interface (synchronous or asynchronous) is not closed; each approach has its advantages and its disadvantages. Significant work is still necessary as well for efficient measure and process systems as for adaptation of interfaces to the severe disabilities in order to democratise BCI systems. To federate and encourage the realization of BCI, various co-operative platforms allow exchange and mutualisation of drivers for measurement systems, signal processing for feature extraction and classification, standard interfaces design. In France, the INRIA developed in partnership with the INSERM and France Telecom R&D the OpenVIBE project [85] which gives access to many software tools for the design of a BCI. In United States, a project called BCI2000 [86] also gives the possibility to reach tools to develop a BCI.

## Performance evaluation

As we saw above, a broad range of human-machine interfaces is available in the laboratories or in the trade making it possible to a person with disability to control a communication and environment control assistive device. An essential difficulty which arises then is the choice of the best interface for a given person. This problem is all the more complex here that the users concerned have physical and cognitive characteristics diverses. Thoughts on the evaluation of these characteristics to contribute to the choice of the assistive device were carried out of long standing [87]. However an evaluation of performance in this context cannot generally dissociate the sensor and the user. Thus we are brought to assess the human-machine system in particular by means of interaction models or of models of the user associated with a model of the task as in [88]. The objective in the particular case of assistive communication is to maximize the flow of information while minimizing the physical and mental workload of the user [89]. In what follows we shall review the methods reported in the literature aiming at measuring the performances of alternative communication systems by distinguishing two cases: use or not of human-machine interaction models.

### Measurements of performance without interaction model

A measurement of performance may be deduced of an experimentation concerning a panel of users with disability, generally compared with a pilot panel made up of people without disability. The question which arises here is the choice of the criteria of performance. Thus to evaluate a tilt sensor intended to emulate a mouse, Chen chooses to measure the accuracy of the pointing, this in a binary way (selections successful or not), as well as the time of realization of the task [90]. These two same parameters, action time and missed selections, are also adopted by Junker et al. for the evaluation of Cyberlink, a human-machine interface using simultaneously EMG and EEG signals [2]. In [91] the authors developed a pointing device (IPDA: Integrated Pointed Device Aparatus) intended for people with tetraplegia, which assigns the pointing and clicking functions of a mouse to different devices and different body parts. The criterion selected to measure its performance, named OE (Operational Efficiency), is defined like the reverse of the task completion time.

In some situations, it is possible to relate a level of motor deficit with the most suitable input sensors. Thus, for people with high-level spinal cord injuries, Bates listed the plausible interface sensors according to the level of the injury classically denoted as Cn, n numbering the cervical branch [92]. For example, an eye tracker device or tongue controlled switches would be candidates for being used for a C1 spinal cord injury level. A chin joystick or shoulders switches could be selected from C3 to C5 whereas the use of EMG signals could be recommended from C1 to C8 by choosing the most suitable muscles.

In [93] the authors assess the ergonomics of an alternative mouse based on EMG signals collected on the *Frontal, Masseter *and *Trapezius *muscles. The objective is, at the same time, to evaluate the ergonomics of the EMG interface and to carry out a comparison between these muscular zones as control inputs. The measurement of performance uses questionnaires and some ergonomic criteria defined for a pointing task: time to reach a target from the previous one, numbers of mouse clicks before reaching a target and number of clicks on the wrong button. Criteria of selection errors and movement times are also used by Chin et al. to evaluate a pointing task in order to compare an EMG interface and an eye-gaze tracking interface [22].

The information theory initially developed for signals transmission in telecommunication [94] is often used to evaluate the human-machine or machine-human transmission channel. It is in particular the case in the field of brain-computer interfaces. The recognition of control information in EEG signals being disturbed with errors, we can define an "Information Transfer Rate" (ITR) by analogy with the capacity of a noisy transmission channel [95,96]. If each item target among N has the same probability p of being selected without error, we are in the case of a symmetrical channel with N symbols whose capacity is given by:(1)

This in particular makes it possible to choose, p being given, an optimal number N of targets to be proposed to the user, i.e. which maximizes C [95]. If we multiply this parameter by the number of commands per second μ, we obtain the bandwidth (or "throughput") TP of a given interface [97]:(2)

Tonet et al. associate to the "throughput" a second parameter, the "latency", defined as the time between the moment when a command is initiated and the moment when its effects start. They thus link the performance of a control interface to the needs of the assistive device [97]. The "Information Transfer Rate" can also be applied to human-machine interfaces based on switch sensors. Thus Huo et al. uses it as criterion of performance for the evaluation of a wireless magneto-inductive sensor controlled by the tongue [98]. The tests relate to a mouse emulation task and show an ITR of 130, superior to that of the BCI interface described by Wolpaw [99] and also to those of other tongue-computer interfaces.

Let us finally note that quantitative performance criteria are not always convenient during an evaluation on a panel of people with disability. Thus Betke and al. measure a completion time to compare a task of text selection on a virtual keyboard using a mouse and then a "camera mouse" (device allowing to carry out a visual tracking of a body feature), this on a panel of people without disability [100]. On the other hand, for their experiments on users with disability, they are satisfied with qualitative data.

### Measurements of performance with interaction models

#### Direct communication

As regards modelling in the field of assistive technology the majority of the studies reported in the literature aim at adapting to people with disabilities a model initially defined for people without disability. Koester and Levine, for example, modelled the user performance for a text entry task with words prediction using a direct control of the keyboard with a headstick [101]. To the usual parameter "Keystroke Saving" (the number of keystrokes saved thanks to the words prediction), which reflects only the motor component of the user activity, the authors substitute a cognitive component with a two parameters model, "Keypress Time" and "List Search Time" derived from the KLM model (Keystroke Level Model) [102]. The experiments carried out starting from this model enable them to conclude that the time saved thanks to the keystroke saving is partly compensated by the time wasted to scan for the adequate word in the proposed list.

Sanger and Henderson model the human-machine interaction in the case of an assistive communication device made up of a touch screen [103]. The objective is to optimize the communication rate (IR) according to the number b of items simultaneously on the screen, of the size w of the icons and of the average number m of items to select to reach a vocabulary element. They define IR for that by:(3)

The entropy, according to the information theory, is the average value of -p(x).log_2 _p(x), x being a vocabulary element and p(x) its probability to be selected. TT(w, b, m) is the time necessary to select one item. It is divided in an action time MT, and a time RT for the choice of the item. RT may be given by the Hicks' law which depends on the logarithm of the number b of possible choices [104]. The authors prefer here to use a linear law to take account of the imperfect knowledge of the interface by the users. The action time MT is modelled classically by the Fitts' law [105], frequently used in the analysis of pointing devices.

#### Pointing Tasks

The Fitts' law defines that the response time is given by:(4)

Where the constants "a" and "b" are empirically determined and ID is the index of difficulty calculated in terms of the distance (D) between the starting point and the centre of the target and width (W) of the target:(5)

Originally designed as a model of human psychomotor behaviour, such law leads to a performance measurement standardized and validated by many researchers, being actually used in the International Standard ISO 9241, Part 9: "Requirements for non-keyboard input devices". The performance index is defined by IP = 1/b or, to combine the parameters "a" and "b" in only one metric, IP = ID/MT ("throughput" expressed in bits/s) [106]. It does not allow however a complete analysis of the pointing movement [107]. Thus to evaluate the performances of an EMG-based interface using the neck muscle EMG signals as well as a head orientation sensor, Williams and Kirsch associate the "throughput" parameter of the Fitts' law to other indicators allowing to analyze more precisely the quality of movement [26]: initial reaction time, effectiveness of the path (variation between the path carried out and the straight line), overshoot (the number of occurrences of the cursor reaching the target then leaving it before the end of the dwell time), mean velocity and direction ratio (evaluation of the capacity of the subject to move the cursor in diagonal compared to horizontal or vertical movements).

Lopresti et al. undertook a study aiming at analyzing if neck movement limitations imply a reduction of performance during the use of a head control sensor [108]. They note in particular that, for a pointing task with this interface, their panel of people without disability satisfied to the Fitts' law contrary to the group of 10 people with disabilities (6 with multiple sclerosis, 3 with sustained cervical, 1 with spinal stenosis). They are then brought to model the performance of these people by more precisely analyzing the movement according to 3 phases:

• the reaction phase where the person perceives the goal and initiates the movement: the subjects with disabilities have average reaction times longer than the subjects without disability;

• The ballistic phase corresponding to the fast movement towards the target: the motor difficulties of the subjects with disabilities result in the presence of several peaks in the profile speed whereas we note only one peak for the subjects without disability;

• The homing phase where the movement is slowed down and controlled better to position on the target. As in the reaction phase the time passed in this phase by the people with disabilities is longer on average than that passed by the people without disability;

To evaluate the performances of an algorithm of adaptive adjustment of head movement sensors sensitivity, these same authors measure three parameters: the accuracy (proportion of icons selected successfully during their experiments on a pointing task), the "throughput" ID/MT and the overshoot (relationship between the distance covered after having exceeded the target and the distance to the target) [109].

In a similar way Tanimoto et al. present an analysis software of the pointing movement which draws the trajectory of the cursor on the screen [110]. For people with tetraplegia they note that this trajectory does not follow a " Fitts' configuration» but is punctuated with stopping periods. Then they characterize it by parameters like the time in the stopping period before the click or the times during the moving and positioning phases, quite similar in their definitions to the ballistic and homing phases described above, as well as the distances covered, the velocities and the stopping times during these two phases.

The Fitts' law, usually used to analyze and evaluate the performances of the pointing tasks, is not verified for some persons with disabilities. Thus Gump et al., after an experimentation relating to 8 subjects with cerebral palsy, conclude that for a majority of these subjects the movement time MT is better represented by a ballistic law (function of the square root of the distance to the target) that by the Fitts' law [111]. The authors explain this by the oculomotor difficulties of the subjects. These conclusions are however partly contradicted in [112]. Here, for the same type of subjects as previously, the authors choose to carry out their experiments on a very simple manual pointing task to be free from cognitive problems which might affect the movement accuracy. This time the Fitts' law is checked in spite of the significant motor difficulties of the subjects.

Felton et al. [113] successfully used the Fitts' to the evaluation of performance on a target acquisition task during sensorimotor rhythm-based BCI training. The participants in the study were both disable and able bodied volunteers but with the inclusion criteria of consistent target acquisition task accuracy exceeding 80%, justified by the fact that the Fitts' law emphasizes time over accuracy. However, a few researches may find that the accuracy should be taken in account in the general evaluation of pointing tasks systems, especially in the case of BCI systems that may present great performance variation within the same session.

Gajos et al. describe a Supple++ software which generates user interfaces automatically adapted to the motor capacities of the person [114]. Observing that the Fitts' law is not always checked for people with disabilities, they propose to model the pointing task in a personalized way. They try for that, for each user, the possible combinations of 7 parameters: a constant term, the index of difficulty ID = log_2 _(1 + D/W) of the Fitts' law (with D the distance to be crossed and W the width of the target), log_2 _(W), log_2 _(D), W, 1/W and D.

#### Scanning systems

The scanning systems controlled by adapted switches, only method usable for many people with severe disability, generate an intrinsically slow communication (about 1 to 2 words per minute). It is thus necessary to optimize the communication rate and this in a personalized way in order to adapt to the great diversity of the physical and cognitive capacities of the users concerned. The parameters which we can adjust for this purpose are summarized by the model initially defined by Rosen and Goodenough-Trepagnier giving the average time T necessary to select a word [115,116]:(6)

Where C is the linguistic cost (the average number of language units per word), function of the selected language (alphabetical, phonemes, etc). L is the average number of actions to select a language unit and *t *is the average time per action. This model initially developed for direct communication devices has been extended to the scanning systems by Damper [117]. The product L.*t *is then a function of the type of scanning, of the geometrical structure of the matrix of items and of the elementary scanning delay. This study does the assumption that, after each selection of item, the scan starts again at the beginning of the matrix. Bhattacharya then extends the model to scannings starting from the selected item [118]. In addition to the probability of selection of each item k_i_, it is necessary in this case to use that of the digraphs (k_i_, k_j_). Moreover, if we want that the performances calculated with the ideal models correspond to those measured in practice, it is necessary to take account of the selection errors which can be numerous with users with severe disabilities. In [119] it is proposed a users errors modelling by classifying them in two categories: timing errors (the user actuates the sensor too late) and selection errors (the user selects a wrong element (block, row or item)).

In the models evoked above, the user performance is reflected by the elementary scanning delay T_scan_. This time is adjusted in an empirical way in the commercialized systems or in an adaptive way in some studies [1,120]. The action on a switch following a visual stimulus (change of color or appearance of an item) may be modelled using the MHP model (Model Human Processor) initially developed by Card, Moran and Newell for computer tasks of low cognitive level (reaction to stimuli) [102]. It requires a time T_act _= T_p _+ T_c _+ T_m _where T_p_, T_c _and T_m _are respectively the elementary perception, cognition and motor times. This model is used in [121] for the design of a simulator aiming at assessing the assistive communication devices. It is also the case in [118] but by adding an additional cognitive time following the study reported in [122]. Keates et al. indeed tested the applicability of the MHP model on a panel of people with disabilities for a simple task: the activation of an adapted switch in reaction to a visual stimulus. They noted, on the one hand, motor times on average higher than those measured on people without disability, and, on the other hand, the presence of an additional cognitive time compared to the theory, corresponding to the decision of releasing the sensor. In [123] it is also noted for the use of a scanning communication device that some persons with disabilities do not conform to the MHP model due to motor disorders (persons with cerebral palsy) or cognitive disorders (persons with cranial trauma). Finally, in [1], it is proposed an improvement of the MHP model applied to scanning communication devices, the "three-zone behaviour model", which takes into account the fact that, very often, the user, accustomed to the scanning rate, starts to react before the visual stimulus appears.

## Conclusions

In the last 20 years, driven by technical advances and government initiatives, assistive devices aimed to AAC increased enormously in number and variety. Access to AAC equipments can be made by means of electrical biosignals, which control is possible even for people with severe motor impairments.

The EMG signal has the advantage of almost instantaneous response, and with four muscles available it is possible to establish control over a cursor. At first, it may not be very intuitive to move a cursor using, for instance, facial muscles. As studies indicate, diagonal movements are usually executed by horizontal and vertical displacements, but with increasingly user ability during training. The major caveat of using muscles is the fatigue that may impede prolonged time of activity. The number of muscles available varies greatly among patients with motor impairments, leading to a vast amount of strategies on using the EMG signal. For users with severe motor impairments it is expected that few muscles can be explored and therefore limiting the applications to scanning systems or cursor control in an unnatural way, as explained before. One possible improvement in the future is to develop signal processing algorithms that allow more reliable progressive signals encoding during contractions, what could improve dramatically performance of EMG-based AAC systems.

The use of the EEG signals has gone by intensive research over the last two decades and has proved to be effective for various applications, as indicated by Wolpaw [3]. The BCI systems present high performance variability and present technical problems that must receive special attention, such as contamination by EMG signals. Additionally, BCI systems are not indicated to everyone, as there are people incapable of efficiently modulate brain waves. But since the head is the last site to suffer degradation in cases of severe disability, its use should be considered in extreme motor impairment conditions.

The EOG signal has two advantages displayed by the two previous biosignals. As the EEG signal, the ability of a person to control biological process associated with the EOG signal is preserved even in extreme situations of motor impairment; and similar to the EMG signal, the EOG signal presents low response time. One of the applications of the EOG signal is cursor control task, although is generally deprecated when compared to gaze-based systems, that also use eye motion but spare the user the necessity of using electrodes attached to the skin. In fact, this last disadvantage is shared with the use of EEG and EMG signals, demanding proper skin preparation and electrodes placement, in what concerns position and possibly orientation. Dry electrodes adoption is an option, with electrical characteristics meeting the needs for recording electrical biosignal suitable to signal processing tools currently in use. The mechanical characteristics in the other hand, present the problem of higher mass when compared to gelled electrodes given the presence of pre-amplifier circuitry due to high electrode-skin impedance.

The choice of the ideal solution for AAC is not trivial. It is well established that rehabilitation technologies deployment demands that each patient is analyzed individually. Unfortunately, there are few commercial devices available that explore electrical biosignals, and it is not probable that this picture is going to change in the short term. One of the reasons is the different degrees that the same condition (e.g. tetraplegia) afflicts different people, demanding not only user training but also user customization. For instance, in [124] the participant in a study for the deployment of a BCI was submitted to a functional magnetic resonance imaging (fMRI) to find the best sites to place electrodes. Beyond commercial approach, successfully adoption of the device depends on several factors, among them the caregiver ability to learn and personalize the new tool [125]. Therefore, it is necessary to close the gap between the knowledge required by the caregivers and the system complexity during set-up, support and training.

Other considerations include aesthetics, as a device aiming for communication is likely to be used during social circumstances. Also, when idealizing the system, it should be considered the user effort in the operation of a device that ideally should be used for several hours a day. Most of the studies however, are conducted with patients only in the Phase I clinical trials, with few sessions per week, proving only the technical viability to transform electrical biosignals into commands, but failing in providing insights about the device operation in daily basis. Finally, assistive devices should have an operation principle as simple as possible. Solutions such as presented in [20] with a single muscle controlling the cursor position in two axes does not seem to be very easy to operate. In this case, the system complexity moved towards the user, which was obligated to execute an apparently hard task to achieve a simple operation.

According to the 1990 U.S Census Bureau's National Health Interview Survey, about one-third of assistive devices not needed for survival are unused or abandoned just 3 months after they were initially acquired [6]. Nevertheless, some efforts of the world's scientific community for trying to change this paradigm and that are cited in this article, are synthesized in Table 1.

**Table 1:** Summary of methods suitable for alternative communication

Method based system	Reference	Description/Application
Mechanical	[1]	Switch device controls scan-based system
	
	[17]	Morse code-based system controlled by sip-and-puff device.
	
	[27]	Head motion detected by a motion sensor allows the user to control a cursor on the screen. Click and double click was performed by the user inflating the cheek and touching the switches.
	
	[90]	Tilt sensors for cursor control.

EMG	[9]	Device control such as wheelchairs, indicating the possibility of being used for AAC purposes.
	
	[11,38-41,43]	Recorded from vocal articulation muscles, EMG signal features are used in the task of speech recognition.
	
	[19]	EMG offers switch-based control signal used in a scanning system.
	
	[18]	Morse code-based systems.
	
	[20,25,28,29,93]	Cursor control/pointing device established by EMG signals recorded from muscles that can be controlled by people with tetraplegia at the C4 level.

EOG	[47,48]	A system for writing in an alphanumeric matrix based on two EOG channels (vertical and horizontal)
	
	[54,56,57]	Cursor control by eye movement direction.
	
	[58]	Eye movements are translated to Morse code symbols to issue command messages.
	
	[59]	Sequences of eye movements are associated to symbols (10 Arabic numerals and 4 arithmetic operators).

EEG	[8,62]	EEG (Mu and Beta rhythms) operate a 1D graphic device.
	
	[63-65,69,70,78-80,82]	Language support controlling spelling systems.
	
	[74]	Cursor control using spike activities detected by implanted electrodes
	
	[71,72,83,84]	Device control such as appliances or a wheelchair, indicating the possibility of being used for AAC purposes

Hybrid systems and others	[2]	Both EEG and EMG signals are applied to cursor control, including click.
	
	[3,12,21]	EMG signals from facial muscles are used to control a cursor in 2D. The EEG signal acts as an ON/OFF switch.
	
	[100]	Using a camera, the system tracks the computer user's movements to control the cursor on the screen.
	
	[13,22,23]	EOG signals define the absolute cursor position on the screen and EMG signals are used for small displacements.
	
	[14,42]	EMG signals from muscles of vocal articulation are used to complement audio signals information in the task of speech recognition.
	
	[24]	Cursor control system with the position controlled by gaze and the object selection activated by frowning.
	
	[30]	Images and EMG signals are used to determine face position that can be used to intent expression.
	
	[49]	Virtual keyboard writer system based on two EOG channels and one EMG channel for letter selection.

If factors such as aesthetics are subjective, the quantitative analysis of an AAC system is an objective and vital step in the choice for a given patient. It was observed that for the same task, i.e. cursor control, different measures are taken, preventing effective comparison of different sensors. The Fitts' law used to measure efficiency of pointing control device is a general accept method, and has even been used in the ISO 9241 norm for device evaluation, despite its adoption should be carefully analyzed as some solutions do not follow a "Fitts' law" approach.

Despite the situation people with severe motor impairments are found, several solutions for AAC are available, exploring last abilities remaining. For instance, one person with tetraplegia at the C2 level may present control only from the neck up. Even though, all of the three biosignals addressed in this article could be explored by this user. Besides the user capability of using a given system, other considerations should be made, such as ergonomics, and also performance. An optimal choice of the control interface of an assistive device supposes an individualized assessment of the human-machine interaction. For that it is often interesting to call upon models of human-machine performance. Those interesting in our context of study are defined for a task or a category of tasks, for example Fitts' law for a pointing task, Hick's law for a choice of alternatives, MHP model for the simple motor reaction to a visual or an auditory stimulus. When we call upon this type of models, well established for people without disability, it is however essential to verify that they are adapted to the motor, perceptive and cognitive capacities of the person with disability concerned.

## Competing interests

The authors declare that they have no competing interests.

## Authors' contributions

PP analysed the study over sensors classification. CGP and AAO analysed current literature on use of EMG signals on alternative communication. ELN was responsible for the EOG section. EL carried out the analysis on EEG. GB was responsible for the section on performance methods. AAO and GB supervised, revised and gave the final approval of the manuscript. All authors read and approved the final manuscript.
